# Time trends in pediatric hand fracture incidence in Malmö, Sweden, 1950–2016

**DOI:** 10.1186/s13018-021-02380-y

**Published:** 2021-04-09

**Authors:** Erika Bergman, Vasileios Lempesis, Lars Jehpsson, Björn E. Rosengren, Magnus K. Karlsson

**Affiliations:** Clinical and Molecular Osteoporosis Research Unit, Department of Clinical Sciences and Orthopedics, Lund University, Skåne University Hospital, SE-205 02 Malmö, Sweden

**Keywords:** Boys, Girls, Children, Fractures, Epidemiology, Time trends, Joinpoint, Etiology, Hand

## Abstract

**Background:**

The hand is the second most fractured region in children. It is therefore important to update fracture epidemiology to be able to identify time trends for adequate health care planning. This study reports pediatric hand fracture incidence 2014–2016 and, using published data, also long-term time trends in 1950–2016.

**Patients and methods:**

The Swedish city of Malmö, with 328,494 inhabitants in 2016, has only one hospital. We used the hospital radiological archive, medical charts, and diagnosis registry to identify hand fractures in city residents < 16 years in 2014–2016. These data were compared to those from three published studies that evaluated periods in 1950–2006. Differences between two periods were calculated as both unadjusted and age- and sex-adjusted incident rate ratios (IRR) with 95% confidence intervals (95% CI). We used joinpoint regression to estimate time trends during the entire period and present annual percent changes (APC) with 95% CI.

**Results:**

In 2014–2016 phalangeal fractures accounted for 71% of all hand fractures, metacarpal fractures for 24%, and carpal fractures for 5%. We identified 615 hand fractures (419 in boys and 196 in girls) during 181,617 person-years in 2014–2016, resulting in an unadjusted pediatric hand fracture incidence of 339/100,000 person-years (boys 452/100,000 person-years and girls 220/100,000 person-years). The age-adjusted incidence 2014–2016 was similar to 2005–2006, the most recently evaluated period (IRR in boys 0.9; 95% CI 0.8 to 1.01, and in girls 1.0; 95% CI 0.8 to 1.2). Looking at the entire period 1950–2016, we found that age-adjusted incidence increased in 1950–1979, in boys by APC + 3.8%; 95% CI 3.0 to 4.5 and in girls by + 3.9%; 95% CI 2.8 to 5.0, but decreased in 1979–2016, in boys by − 0.7%; 95% CI − 1.4 to − 0.003, and girls by − 1.3%; 95% CI − 2.4 to − 0.1.

**Conclusions:**

Phalangeal fractures accounted for about three quarters of all hand fractures. The age-adjusted hand fracture incidence increased in both sexes in 1950–1979 and decreased in 1979–2016.

**Level of evidence:**

III

**Supplementary Information:**

The online version contains supplementary material available at 10.1186/s13018-021-02380-y.

## Background

Some 30% of boys and 19% of girls have sustained a fracture before the age of 18 [1]. Distal forearm fractures comprise one fourth to one third of these, and hand fractures (phalangeal fractures, metacarpal fractures, and carpal fractures) about 17–24% [2–4]. However, fracture incidence may be exposed to time trends, as studies from our region have identified higher age- and sex-adjusted hand fracture incidence 1976–1979 than 1950/1955, but similar incidences 1976–1979, 1993–1994, and 2005–2006 [5]. Studies from many settings report time trends: from Finland with lower pediatric hand fracture incidence in 2005 than in 1983 [4], from the USA with decreasing fall-related hand/finger fracture incidence between 2001 and 2015 [6], and from Denmark with lower incidence of metacarpal fractures in age groups 6–11 and 12–15 in 2015–2018 than in 1994–1999 [7]. Time trends may be different in different regions and during different periods but at least part of the differences may be due to methodological issues, such as different ascertainment methods, or that inferences are based on comparison between absolute incidences or age- and sex-adjusted incidences. To address these shortcomings, region-specific examinations of pediatric hand fracture incidences are needed using the same ascertainment method over time and with adjustment for changes in demographics. This will make it possible to identify time trends and improve the allocation of adequate health care resources.

As there are indications of time trends also in fracture etiology [3, 5, 8, 9] any examination should also include this. In Sweden, for example, we have found indications that structured traffic safety strategies as well as home environment injury protection seem to have influenced the incidence of traumatic injuries in children [10]. Updated etiology data could be used to evaluate the effectiveness of fracture preventive strategies and to identify new risk activities for fracture and new prevention demands.

The aims of this study were to evaluate pediatric hand fracture epidemiology/etiology in Malmö, Sweden, in 2014–2016 and, using published data [3, 5, 8, 9], to evaluate time trends in pediatric hand fracture epidemiology/etiology 1950–2016.

## Patients and methods

Malmö is the third largest city in Sweden, with a population of 328,494 in 2016 (62,513 under 16 years of age) [11]. The city has only one hospital, which provides trauma care for the city residents. At the hospital, all radiographs, referrals, and medical charts have been saved in an archive for a century, making it possible to conduct retrospective epidemiological studies and to collect patient-specific data [3, 12]. Until 2001, the radiographs in the archive were classified according to diagnosis, anatomical region, and year of injury and saved as analog radiographs. The archive has been used to describe pediatric fracture data for the years 1950, 1955, 1960, 1965, 1970, 1975–1979 [8], and 1993–1994 [9].

The Department of Radiology changed from analog to digital radiographs in 2001. Since then, the radiographs have been classified according to the patient’s individual personal identity number in a digital archive. This archive was used in 2005–2006 [3, 5] as well as in the current study. To identify fractures, we searched, as was done in 2005–2006, the digital in- and outpatient diagnosis records at four departments (Emergency Department, Department of Orthopedics, Department of Hand Surgery, and Department of Otorhinolaryngology) [3, 5]. The records had to fulfill the following three criteria to be included: (I) any of the Swedish version of the International Statistical Classification of Diseases and Related Health Problems 10th revision (ICD-10-SE) fracture diagnosis codes S02.3–S02.4, S02.6–S02.9, S12.0–S12.2, S12.7, S22.0–S22.1, S32.0–S32.8, S42.0–S42.9, S52.0–S52.9, S62.0–S62.8, S72.0–S72.9, S82.0–S82.9, or S92.0–S92.9; (II) patient age 0–15 years at fracture event; and (III) Malmö city residency at fracture event. During 2014–2016, we identified a total of 7326 visits, of which 1632 concerned a hand fracture (ICD-code S62.0–62.8; phalangeal fracture, metacarpal fracture, and carpal fracture). To verify fractures found by this ascertainment method, we reviewed medical charts, referrals, radiographic reports, and radiographs. This made it possible to validate the fracture diagnosis and avoid double counting of fractures.

Two different registration protocols were used in our study to register hand fractures. In the first protocol, fracture registration was done using the same registration protocol as in preceding studies [3, 5, 8, 9]. Hand fractures are categorized into three different groups: (i) phalangeal fractures, (ii) metacarpal fractures or carpal fractures, and (iii) scaphoid fractures. Multiple fractures in the phalangeal bones sustained during the same fracture event are, as in previous evaluations, registered as a single fracture; the same applies to multiple fractures in the metacarpal and/or carpal bones. Fractures in more than one of the groups listed above, bilateral fractures and refractures are classified as separate fractures. Traumatic amputations are not classified as a fracture. We used this classification system to be able to compare results from 2014 to 2016 with those from 1950 to 2006.

In the second registration protocol, used in the most recently published study [5], we registered multiple phalangeal fractures, multiple metacarpal fractures, multiple carpal fractures, and combinations of metacarpal and carpal fractures as separate fractures. This protocol facilitates reporting of anatomical distribution of hand fractures in detail, and comparison to other reports in the literature, which have recorded hand fractures in this way [13–19].

As in the previous studies [3, 5, 8, 9], we registered fracture information regarding age at fracture event, sex, fracture date, fracture location/locations, fracture side, trauma mechanism, trauma severity, and trauma activity. The severity of the trauma was classified into slight (falls below 0.5 m, and most of the sports injuries), moderate (falls from 0.5–3 m, falls from swings and slides, and bicycle injuries), or severe (falls from more than 3 m, and motor traffic injuries). We also classified fracture etiology according to the NOMESCO Classification of External Causes of Injuries (NCECI) [20]. This classification enabled us to collect information on both location and activity, in contrast to the Landin classification [3, 5, 8, 9].

To validate the fracture ascertainment method, one of the authors (VL) searched the digital in- and outpatient diagnosis records during 2 months (1 January to 28 February 2005). By this, 103 fractures were identified in Malmö children < 17 years. A review of the digital radiological archive at the Department of Radiology with the same search criteria also identified 103 fractures. Both methods identified the same 100 unique fractures. Each of the methods also identified three other fractures which were not identified by the other method. This resulted in a total of 106 fractures, of which three were missed by each method. This corresponds to a misclassification rate of 3% [3].

Microsoft Excel 2016 and SPSS Statistics 26 were utilized for database management and statistical calculations. Data are presented as the number of fractures, proportions (%), and incidences per 100,000 person-years (data on population at risk were acquired from official population records [11]). Age- and sex-adjusted incidences were estimated by direct standardization with the average pediatric population in the city (in 1-year classes) during the examined period as reference. Due to lacking information on etiology for many fractures, we present only descriptive etiology data. We grouped the examined years into six decades (1950/1955, 1960/1965, 1970/1975–1979 [8], 1993–1994 [9], 2005–2006 [3], and 2014–2016) and then compared fracture incidences between two decades by incident rate ratio (IRR) with 95% confidence interval (95% CI) to describe the uncertainty. We finally estimated time trends for the entire period 1950–2016 using joinpoint regression. This analysis, in contrast to IRR, takes the variation between years/periods into consideration, and we used data for each of the 17 estimated years. Time trends are presented as annual percent changes (APC) with 95% confidence intervals (95% CI) [21, 22]. A *p* value below 0.05 was considered to represent a statistically significant difference.

## Results

### Fracture epidemiology 2014–2016 (multiple fractures classified as separate fractures)

**Fig. 1:**
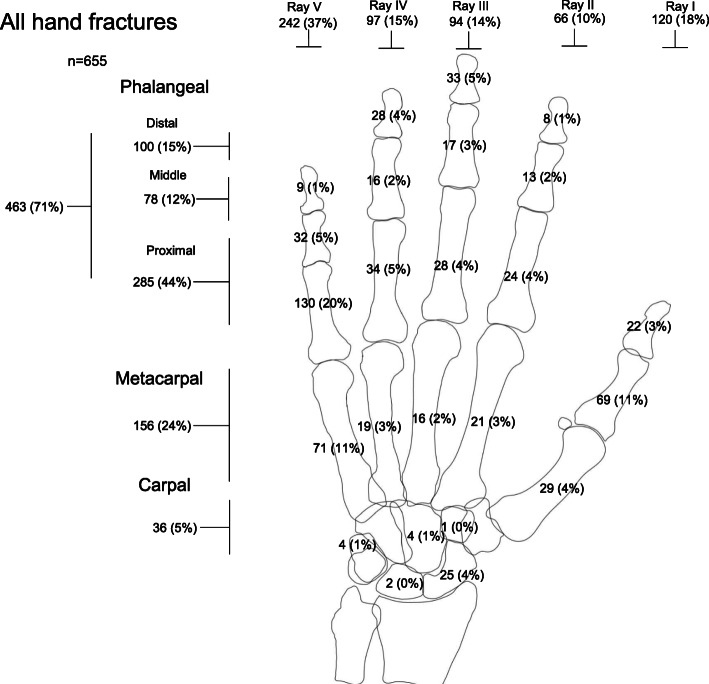
The anatomical distribution of hand fractures in children 0–15 years, in Malmö, Sweden, in 2014–2016, presented as numbers with proportions of all hand fractures in brackets. The sum for each ray (metacarpal and phalangeal fractures) is presented above the respective ray and the sums of phalangeal fractures (distal, middle, and proximal phalangeal fractures), metacarpal fractures, and carpal fractures are presented on the left

With this classification, we included 655 pediatric hand fractures during the 3 evaluated years. Of these, 71% were phalangeal, 24% metacarpal, and 5% carpal fractures (Fig. 1). Data on distribution of hand fractures in boys and girls and in the left and right hand are presented as Supplement Figure 1 and 2.

### Fracture epidemiology 2014–2016 (multiple fractures classified as one fracture, in agreement with historic studies from our city [3, 5, 8, 9])

By the Landin classification system, we included 615 hand fractures (419 in boys, 196 in girls) out of total 3244 pediatric fractures during 181,617 person-years. Hand fractures were the second most common pediatric fracture (19% of all fractures) after distal forearm fractures (31% of all fractures) [23]. Hand fractures in children had an incidence of 339/100,000 person-years (452/100,000 person-years in boys and 220/100,000 person-years in girls). There were 440 (274 in boys and 166 in girls) phalangeal fractures in all children, resulting in an incidence of 242/100,000 person-years (296/100,000 person-years in boys and 187/100,000 person-years in girls). In all children, 150 (127 in boys and 23 in girls) were metacarpal/carpal fractures with an incidence of 83/100,000 person-years (137/100,000 person-years in boys and 26/100,000 person-years in girls). Twenty-five fractures in all children (18 in boys and 7 in girls) were in the scaphoid bone, resulting in an incidence of 14/100,000 person-years (19/100,000 person-years in boys and 8/100,000 person-years in girls). Boys had a higher age-adjusted hand fracture incidence than girls (IRR 2.2 95%; CI 1.9 to 2.6), but also phalangeal fractures (IRR 1.6 95%; CI 1.4 to 2.0), metacarpal/carpal fractures (IRR 5.8 95%; CI 3.8 to 8.6) and scaphoid fractures (IRR 2.4 95%; CI 1.1 to 5.3) were more common in boys.

**Fig. 2:**
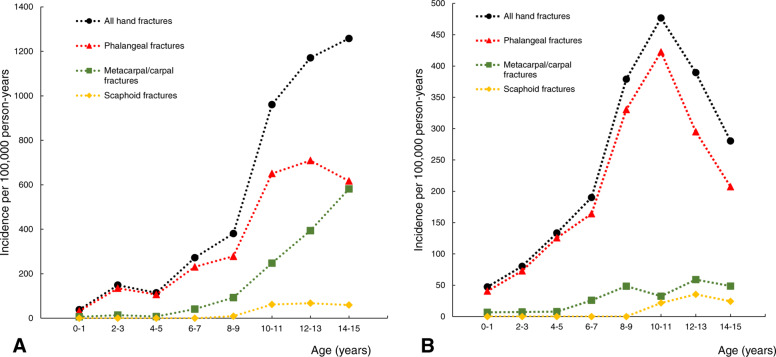
Hand fracture incidence per 100,000 person-years (divided into all hand fractures, phalangeal fractures, metacarpal/carpal fractures, and scaphoid fractures) in boys (**a**) and girls (**b**) aged 0–15 years (combined in 2-years age classes) in Malmö, Sweden, in 2014–2016

The peak hand fracture incidence in boys occurred at age 14–15 and in girls at age 10–11 (Fig. 2) in the evaluated age span of 0–15 years. Peak incidence for phalangeal fractures, metacarpal/carpal fractures, and scaphoid fractures in boys and girls is also shown in Fig. 2. We found no right to left side preponderance in all children for hand fractures, or for phalangeal fractures, while metacarpal/carpal fractures were more common in the right hand (Supplement Table 1). Scaphoid fractures were not examined due to a low number of fractures. Hand fracture incidence was highest in September (48/100,000 person-years) and lowest in July (16/100,000 person-years) (Supplement Figure 3). The seasonal variation in incidence of phalangeal fractures, metacarpal/carpal fractures, and scaphoid fractures is also shown in Supplement Figure 3.

### Comparison of fracture epidemiology between periods in 1950–2016

**Fig. 3:**
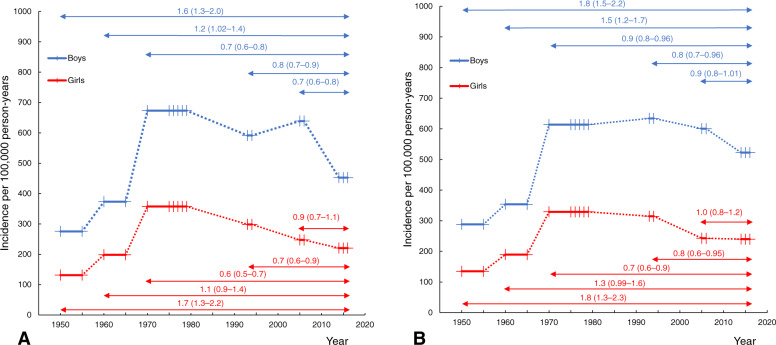
Unadjusted (**a**) and age-adjusted (**b**) hand fracture incidence per 100,000 in Malmö, Sweden, in boys and girls < 16 years of age during the years 2014–2016 in comparison with previous examined periods; 1950/1955, 1960/1965, 1970/1975–1979, 1993–1994, and 2005–2006. The thick lines show the different periods studied and the thin vertical lines within indicate individual study years included in that period. Arrows indicate the period compared with 2014–2016, and the number above shows its associated incident rate ratio (IRR) with 95% confidence interval (95% CI)

**Fig. 4:**
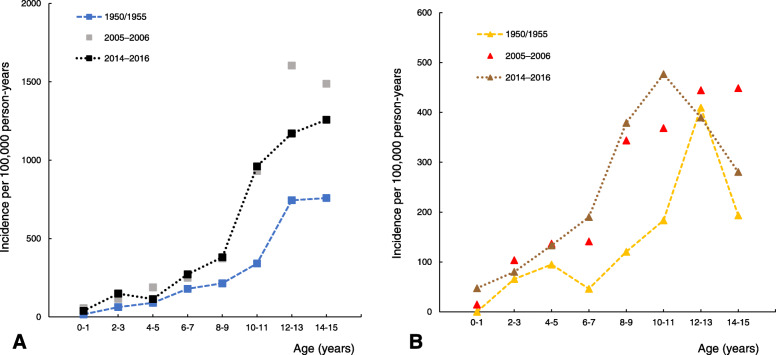
Hand fracture incidence per 100,000 person-years in boys (**a**) and girls (**b**) separately aged 0–15 years (combined in 2-years age classes) in Malmö, Sweden, in 1950/1955, 2005–2006, and 2014–2016

The unadjusted and age- and sex-adjusted pediatric hand fracture incidences in 2014–2016 were higher than in 1950/1955 and 1960/1965 but lower than in 1970/1975–1979 and 1993–1994 (Table 1). Age- and sex-adjusted incidence in 2014–2016 was similar to the most recently evaluated period in Malmö (2005–2006). Sex-specific IRRs for comparisons between different decades are presented in Fig. 3. The corresponding comparisons between decades for phalangeal fractures and metacarpal/carpal fractures are presented in Supplement Tables 2 and 3. Scaphoid fractures were not examined due to a low number of fractures. Sex-specific peak incidences, in the age span of 0–15 years, during different periods are presented in Fig. 4.

**Table 1:** Unadjusted and age- and sex-adjusted hand fracture incidence differences in all children aged < 16 years in Malmö, Sweden, during the years 2014–2016 compared with 1950/1955, 1960/1965, 1970/1975–1979, 1993–1994, and 2005–2006. Data are presented as incident rate ratio (IRR) with 95% confidence interval (95% CI)

Nominator	2014–2016	2014–2016	2014–2016	2014–2016	2014–2016
Denominator	1950/1955	1960/1965	1970/1975–1979	1993–1994	2005–2006
Unadjusted	1.7 (1.4 to 1.9)	1.2 (1.02 to 1.4)	0.7 (0.6 to 0.7)	0.8 (0.7 to 0.9)	0.8 (0.7 to 0.9)
Adjusted	1.8 (1.5 to 2.1)	1.4 (1.2 to 1.6)	0.8 (0.7 to 0.9)	0.8 (0.7 to 0.9)	0.9 (0.8 to 1.02)

### Time trends in fracture epidemiology between 1950 and 2016

**Fig. 5:**
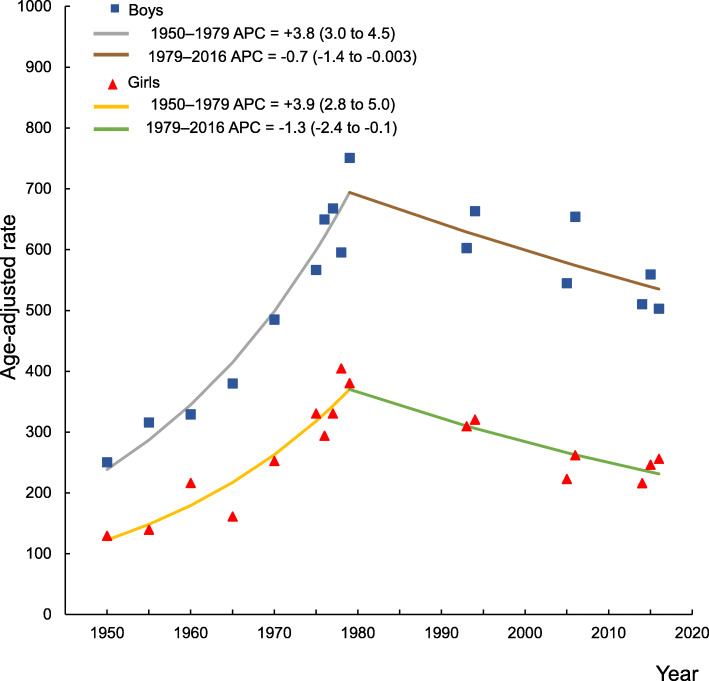
Time trends in hand fractures in the period 1950–2016 calculated by joinpoint regression. Age-adjusted hand fracture incidence per 100,000 person-years in Malmö, Sweden, in boys and girls < 16 years. Data are presented as annual percent changes (APC) with 95% confidence interval (95% CI) to describe uncertainty

The age-adjusted pediatric hand fracture incidence increased during the period 1950–1979 in boys by APC + 3.8%; 95% CI 3.0 to 4.5 and in girls by + 3.9%; 95% CI 2.8 to 5.0. The incidence decreased during 1979–2016 in boys by − 0.7%; 95% − 1.4 to − 0.003 and in girls by − 1.3%; 95% − 2.4 to − 0.1 (Fig. 5).

### Fracture etiology 2014–2016

Most hand fractures occurred during sports activity, followed by activities at school, and playing activity (Table 2). By the NCECI classification system, when fractures with unknown etiology were removed, 75% of fractures in school resulted from sporting activities, and 14% from playing activities (Table 3). Sporting activities accounted for 60% of known fracture data and playing activities for 25%. The most common trauma mechanism was mechanical force, and slight trauma was the most common trauma severity (Table 2). The etiology distribution according to Landin’s classifications system for phalangeal fractures, and metacarpal/carpal fractures is reported in Supplement Tables 4 and 5. We chose not to report the etiology of scaphoid fractures due to few fractures.

**Table 2:** All hand fracture etiology in Malmö children < 16 years during six periods; 1950/1955, 1960/1965, 1970/1975–1979, 1993–1994, 2005–2006, and 2014–2016. Etiology is described as trauma activity, trauma mechanism, and trauma severity. Data are presented as proportions (%) of known trauma etiology

	1950/1955	1960/1965	1970/1975–1979	1993–1994	2005–2006	2014–2016
**Trauma activity**						
**Known**	**42**	**52**	**58**	**72**	**70**	**75**
**Unknown**	**58**	**48**	**42**	**28**	**30**	**25**
**Home**	**10**	**6**	**5**	**7**	**1**	**4**
**Day nursery**	**0**	**0**	**0**	**2**	**1**	**5**
**School**	**15**	**10**	**6**	**5**	**10**	**20**
**Work**	**4**	**0**	**0**	**2**	**0**	**0**
**Traffic injuries**	**21**	**16**	**14**	**19**	**13**	**8**
Bicycle	19	8	9	14	12	7
Pedestrian hit by vehicle	1	5	1	0	0	0
Moped, motorcycle	0	2	2	2	1	0
Car passenger	1	1	3	1	0	2
Other	0	1	0	2	0	0
**Playing injuries**	**12**	**22**	**14**	**15**	**11**	**15**
Playground	1	2	1	2	2	2
In-lines, skateboard	0	0	1	2	2	3
Sledge, other “snow”	0	0	1	1	1	1
Other	11	20	10	9	6	10
**Sport injuries**	**27**	**34**	**45**	**40**	**42**	**42**
Ball-game	20	27	33	26	30	34
Ice-hockey, skating	4	4	4	3	3	1
Gymnastics and athletics	0	0	1	3	0	2
Horse injuries	4	1	3	3	2	1
Wrestling, boxing, etc. “Contact sport”	0	1	1	3	3	3
Skiing	0	1	3	2	2	1
Other	0	1	0	1	2	1
**Fights**	**10**	**12**	**13**	**11**	**20**	**4**
**Other**	**1**	**0**	**2**	**1**	**0**	**2**
**Trauma mechanism**						
**Known**	**85**	**90**	**95**	**93**	**100**	**94**
**Unknown**	**15**	**10**	**5**	**7**	**0**	**6**
**Falls**	**55**	**59**	**68**	**37**	**41**	**36**
On the same plane	46	49	59	22	29	26
Between planes	9	10	10	15	12	10
**Mechanical force**	**44**	**41**	**32**	**56**	**53**	**61**
**Non-classifiable**	**1**	**0**	**0**	**7**	**6**	**3**
**Trauma severity**						
**Known**	**90**	**91**	**96**	**98**	**99**	**97**
**Unknown**	**10**	**9**	**4**	**2**	**1**	**3**
**Slight**	**67**	**67**	**77**	**76**	**77**	**69**
**Moderate**	**12**	**9**	**9**	**19**	**12**	**8**
**Severe**	**4**	**4**	**3**	**4**	**0**	**1**
**Non-classifiable**	**16**	**20**	**12**	**1**	**10**	**23**

**Table 3:** All hand fracture etiology according to the NOMESCO Classification of External Causes of Injuries in children aged 0–15 years in Malmö, Sweden, in 2014–2016. Fracture etiology is presented as proportions (%) of known trauma etiology and presented as activity that resulted in a fracture in a defined location

Activity	Location						
	Home	Day care	School	Sports area	Playing area	Other	Unknown
Known	33	43	54	93	100	100	33
Unknown	67	57	46	7	0	0	67
Sporting activities	14	0	75	99	0	0	3
Playing activities	71	100	14	1	100	6	71
Other activities	14	0	11	0	0	94	26
Total	100	100	100	100	100	100	100

### Fracture etiology between 1950 and 2016

Sports injuries were the most common cause of fracture in all the evaluated time periods (Table 2). The most common trauma mechanism from the 1950s to the 1970s was fall and from the 1990s to the 2010s mechanical force. In all the evaluated periods, the most common trauma severity was slight injury (Table 2). Trauma etiology between 1950 and 2016 in phalangeal fractures and in metacarpal/carpal fractures is shown in Supplement Tables 4 and 5. We chose not to report the etiology of scaphoid fractures due to few fractures.

## Discussion

The hand is the second most common fracture location in children (after the distal forearm) and about three quarters of hand fractures are phalangeal fractures. Hand fractures are most often caused by sport injuries, with a higher incidence in boys than girls. This study supports other publications, inferring that most hand fractures occur in the fifth ray [13–19], that there is no side preponderance for hand fractures [14, 18], and that peak incidence in hand fractures occurs earlier in girls than in boys [18, 19, 24]. Out of all traffic accidents, bicycle injuries were the most common cause of fracture during all evaluated time periods, also found in the literature [25, 26].

The pediatric hand fracture incidence that we found in 2014–2016 (339/100,000 person-years) was similar to a report from Finland from 2005 (344/100,000 person-years) [4], lower than an incidence from northern Sweden in 2006–2007 (389/100,000 person-years) [2], lower than a reported incidence from the UK in 2000 (418/100,000 person-years) [27], lower than an incidence from Scotland in 2000 (489/100,000 person-years) [28], but higher than an incidence from Canada in 1996–2001 (24/100,000 person-years) [16] and higher than a reported incidence from Greece in 1996–1998 (224/100,000 person-years) [29]. The differences may be due to study-specific methodological issues such as ascertainment methods, age spans, and periods. However, it is important to acknowledge that some of the differences between studies may reflect discrepancies in climate, proportion of rural/urban population, and different proportions of immigrants, factors that may affect the pediatric fracture incidence [1, 30, 31].

We found lower unadjusted pediatric hand fracture incidence in 2014–2016 than in 2005–2006, while the age- and sex-adjusted incidence was similar between these periods. This indicates that the changes in unadjusted incidences were dependent on demographic changes between the two periods. As the age- and sex-adjusted incidence was similar in 2014–2016 and 2005–2006, we speculate that incidence has reached a plateau, indicating the need to continue to follow the pediatric hand fracture incidence. However, inferences on time trends should be drawn with care when comparing IRR between two periods, since such estimations do not take variations between years into account. For example, if we had chosen the year 1970 to represent the decade 1970–1979, the conclusion would have been that the unadjusted incidence in 2005 was 8% higher than in the former decade. Had we instead chosen the year 1979, the conclusion would have been that the unadjusted incidence in 2005 was 35% lower. The advantage of the joinpoint regression analysis is that this method takes variations between years into account when estimating time trends. By this method, we could see that the incidence increased from 1950 onwards, reaching a peak in 1979, whereafter a decrease until 2016 was found in both sexes.

We can only speculate on the reasons for the increase in pediatric hand fracture incidence in 1950–1979 and the decrease in 1979–2016. During these decades there were changes in society that could have affected the pediatric fracture incidences. These include a decline in physical activity in Swedish children since the beginning of the millennium [32], with more time spend in front of screens [33]. This could influence the fracture incidence, as reports infer the risk for fracture is higher in less physically active children than in physically active children [34]. A more sedentary life-style has for example been put forward as one possible explanation why scaphoid fracture incidence in men aged 20–24 years has decreased [35]. The increasing proportion of immigrants in Malmö during recent decades may also have affected time trends, as ethnicity is a factor known to be associated with fracture risk [1, 36, 37]. Furthermore, improved safety strategies in traffic and the home environment may also have influenced the incidence of traumatic injuries [10]. We also speculate that certain activities with high fracture risk may have decreased or increased during the evaluated years. Changes in interest in, for example, contact sports, trampolines, skateboards, and roller blades may influence time trends in pediatric fracture incidence.

However, we cannot exclude that other factors could have influenced our results. For example, it is not unlikely that, with the expansion of the health care sector, patients have become increasingly prone to seek health care even for minor injuries, thereby also increasing the possibility to identify fractures. It could also be that physicians decades ago were less prone to refer children with minor trauma to radiographic examinations, thereby failing to identify these fractures. The removal of the patient fee for X-rays in 1970 could also have affected the patients’ desire to undergo X-ray after a minor trauma [8].

Study strengths include the inclusion of objectively verified fractures without double counting of fractures. The long examination period, more than 60 years with 17 different evaluated years, is a strength in the examination of time trends. These data made it possible to analyze time trends throughout the entire period of examination by joinpoint regression and thereby taking variation between individual years into account. The use of the NCECI system is another study strength, as this system allows registration of both trauma location and trauma, not possible by the Landin classification. Weaknesses include the use of two ascertainment methods, originating from changes in the radiographic archive system of the hospital. However, validation revealed that misclassification rates were low for both methods [3, 38]. Another weakness is that we cannot exclude that some children, living in Malmö but treated for their fracture elsewhere, were missed. However, as the tradition in Sweden is that fractured patients are referred to their home hospital for follow-up, most of these fractures would be identified at this second visit. It would also have been advantageous to have a larger sample size, enabling analyses of small subgroups, for example scaphoid fractures, and minimizing the risk of type II errors. The high proportion of fractures with unknown etiology is another weakness and the reason why we only report descriptive etiology data without using inferential statistics.

## Conclusions

Most hand fractures occur in the phalanges, followed by fractures in metacarpals. Hand fracture incidence increased in both sexes in 1950–1979 but has subsequently decreased. We found similar age- and sex-adjusted incidences between the two latest evaluated periods, in 2014–2016 and 2005–2006. This raises the question whether pediatric hand fracture incidence has reached a plateau and highlights the need to continue to follow pediatric hand fracture incidence in the future, to be able to adequately plan resources for these injuries.

## Supplementary Information

**Additional file 1: Supplement Figure 1.** The anatomical distribution of hand fractures in boys and girls 0–15 years, in Malmö, Sweden, in 2014–2016, presented as numbers with proportions of all hand fractures in brackets. The sum for each ray (phalangeal and metacarpal fractures) is presented above the respective ray and the sums of phalangeal fractures (distal, middle, and proximal phalangeal fractures), metacarpal fractures, and carpal fractures are presented on the left. (PPTX 287 kb)

**Additional file 2: Supplement Figure 2.** The anatomical distribution of hand fractures in left and right hand in children 0–15 years, in Malmö, Sweden, in 2014–2016, presented as numbers with proportions of all hand fractures in brackets. The sum for each ray (phalangeal and metacarpal fractures) is presented above the respective ray and the sums of phalangeal fractures (distal, middle, and proximal phalangeal fractures), metacarpal fractures, and carpal fractures are presented on the left and right side. (PPTX 287 kb)

**Additional file 3: Supplement Figure 3.** Seasonal variation in hand fracture incidence per 100,000 person-years (divided into all hand fractures, phalangeal fractures, metacarpal/carpal fractures and scaphoid fractures) in Malmö, Sweden, in 2014–2016.

**Additional file 4: Supplement Table 1.** Right to left distribution in all hand fractures, phalangeal fractures, and metacarpal/carpal fractures (excluding the scaphoid bone) in all children, and in boys and girls < 16 years in Malmö, Sweden, during 2014–2016. Data are presented as incident rate ratio (IRR) with 95% confidence interval (95% CI). The scaphoid fractures were not examined due to the low number of fractures.

**Additional file 5: Supplement Table 2.** Unadjusted and age- and sex-adjusted phalangeal fracture incidence differences in all children and unadjusted and age-adjusted fracture incidence in boys and girls separately in children 0–15 years in Malmö, Sweden, during the years 2014–2016 compared with 1950/1955, 1960/1965, 1970/1975–1979, 1993–1994, and 2005–2006. Data are presented as incident rate ratio (IRR) with 95% confidence interval (95% CI).

**Additional file 6: Supplement Table 3.** Unadjusted and age- and sex-adjusted metacarpal/carpal fracture (excluding the scaphoid bone) incidence differences in all children and unadjusted and age-adjusted fracture incidence in boys and girls separately in children < 16 years in Malmö, Sweden, during the years 2014–2016 compared with 1950/1955, 1960/1965, 1970/1975–1979, 1993–1994, and 2005–2006. Data are presented as incident rate ratio (IRR) with 95% confidence interval (95% CI).

**Additional file 7: Supplement Table 4.** Phalangeal fracture etiology in Malmö children 0–15 years during six periods; 1950/1955, 1960/1965, 1970/1975–1979, 1993–1994, 2005–2006, and 2014–2016. Etiology is described as trauma activity, trauma mechanism, and trauma severity. Data are presented as proportions (%) of known trauma etiology.

**Additional file 8: Supplement Table 5.** Metacarpal/carpal fracture etiology (excluding the scaphoid bone) in Malmö children < 16 years during six periods; 1950/1955, 1960/1965, 1970/1975–1979, 1993–1994, 2005–2006, and 2014–2016. Etiology is described as trauma activity, trauma mechanism, and trauma severity. Data are presented as proportions (%) of known trauma etiology.

## Data Availability

The datasets used and/or analyzed during the current study are available from the corresponding author on reasonable request.

## Abbreviations

ALF: Avtal om Läkarutbildning och Forskning (Agreement on Compensation for Medical Education and Research)
APC: Annual percent change
CI: Confidence interval
ICD-10-SE: International Statistical Classification of Diseases and Related Health Problems - Tenth Revision - Swedish Version
IRR: Incident rate ratio
NCECI: NOMESCO (Nordic Medico-Statistical Committee) Classification of External Causes of Injuries
SPSS: Statistical Package for the Social Sciences
