# Supplementary material for: Time trends in pediatric hand fracture incidence in Malmö, Sweden, 1950–2016
Source: J Orthop Surg Res. 2021 Apr 9;16:245. doi: 10.1186/s13018-021-02380-y (PMC8034127; doi:10.1186/s13018-021-02380-y)
Supplement: Supplementary file 1 — Additional file 1: Supplement Figure 1. The anatomical distribution of hand fractures in boys and girls 0–15 years, in Malmö, Sweden, in 2014–2016, presented as numbers with proportions of all hand fractures in brackets. The sum for each ray (phalangeal and metacarpal fractures) is presented above the respective ray and the sums of phalangeal fractures (distal, middle, and proximal phalangeal fractures), metacarpal fractures, and carpal fractures are presented on the left. (PPTX 287 kb) [file 13018_2021_2380_MOESM1_ESM.pptx]

## Slide 1
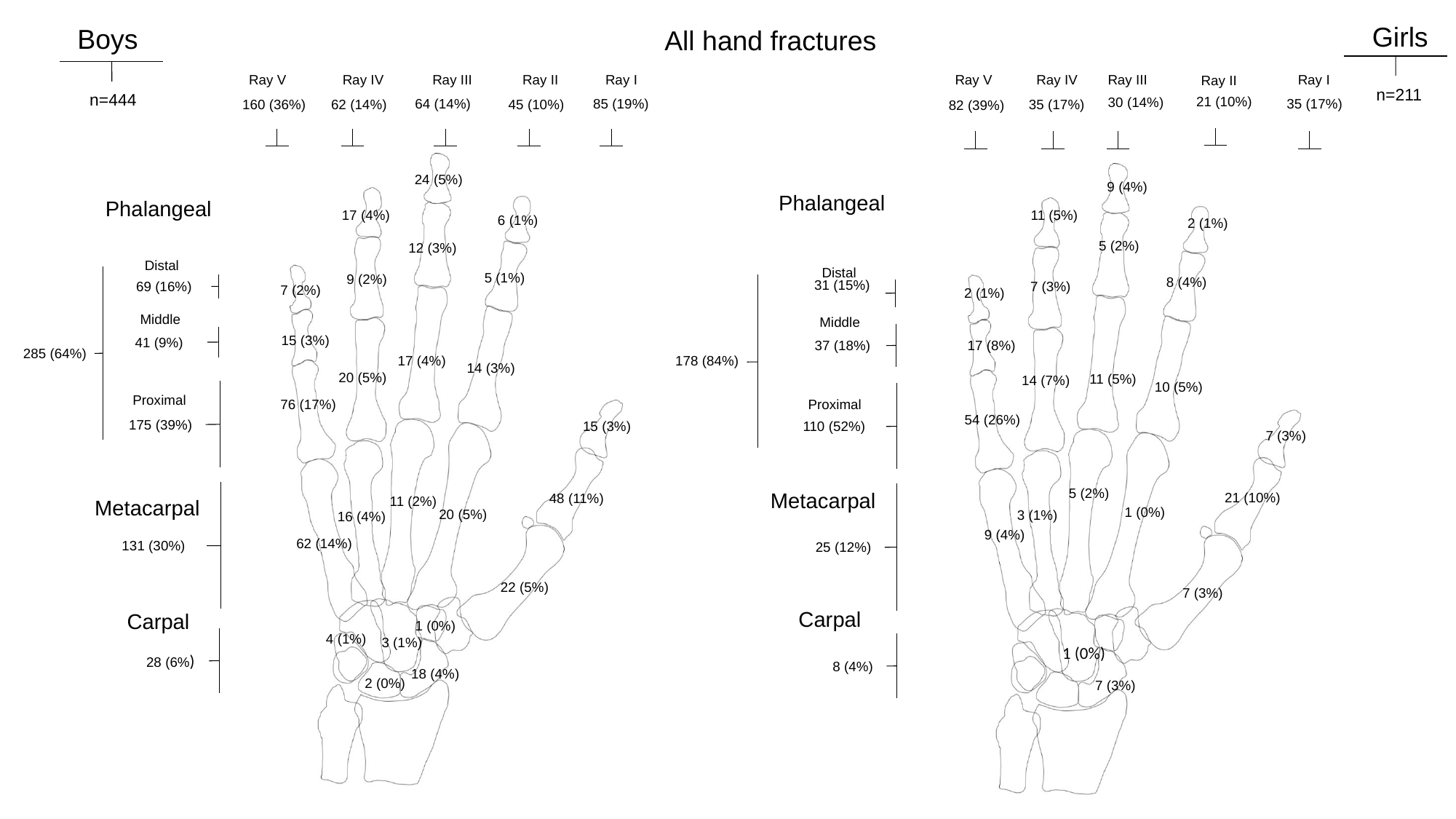

Girls
Boys
All hand fractures
Ray I
Ray V
Ray IV
Ray III
Ray II
Ray V
Ray IV
Ray III
Ray I
Ray II
n=211
n=444
21 (10%)
30 (14%)
35 (17%)
35 (17%)
82 (39%)
9 (4%)
11 (5%)
2 (1%)
5 (2%)
8 (4%)
7 (3%)
2 (1%)
31 (15%)
37 (18%)
17 (8%)
11 (5%)
14 (7%)
10 (5%)
110 (52%)
54 (26%)
7 (3%)
5 (2%)
25 (12%)
21 (10%)
1 (0%)
3 (1%)
9 (4%)
7 (3%)
7 (3%)
64 (14%)
85 (19%)
62 (14%)
45 (10%)
160 (36%)
24 (5%)
17 (4%)
6 (1%)
12 (3%)
5 (1%)
9 (2%)
69 (16%)
7 (2%)
15 (3%)
41 (9%)
17 (4%)
14 (3%)
20 (5%)
175 (39%)
76 (17%)
15 (3%)
48 (11%)
11 (2%)
20 (5%)
16 (4%)
62 (14%)
22 (5%)
18 (4%)
Phalangeal
Phalangeal
Distal
Distal
Middle
Middle
285 (64%)
178 (84%)
Proximal
Proximal
Metacarpal
131 (30%)
Metacarpal
Carpal
Carpal
1 (0%)
4 (1%)
28 (6%)
3 (1%)
8 (4%)
1 (0%)
2 (0%)
