# Supplementary material for: Time trends in pediatric hand fracture incidence in Malmö, Sweden, 1950–2016
Source: J Orthop Surg Res. 2021 Apr 9;16:245. doi: 10.1186/s13018-021-02380-y (PMC8034127; doi:10.1186/s13018-021-02380-y)
Supplement: Supplementary file 2 — Additional file 2: Supplement Figure 2. The anatomical distribution of hand fractures in left and right hand in children 0–15 years, in Malmö, Sweden, in 2014–2016, presented as numbers with proportions of all hand fractures in brackets. The sum for each ray (phalangeal and metacarpal fractures) is presented above the respective ray and the sums of phalangeal fractures (distal, middle, and proximal phalangeal fractures), metacarpal fractures, and carpal fractures are presented on the left and right side. (PPTX 287 kb) [file 13018_2021_2380_MOESM2_ESM.pptx]

## Slide 1
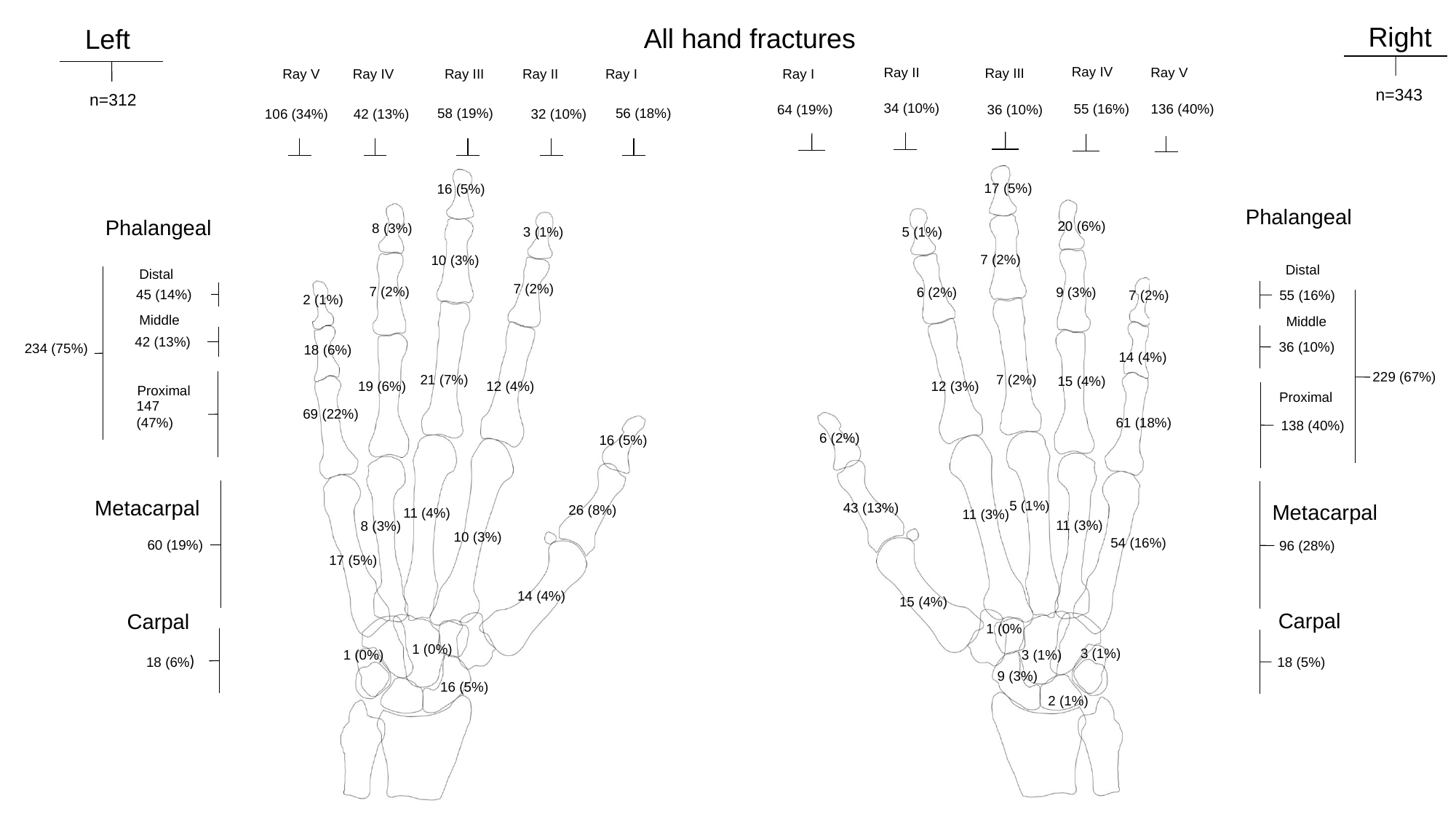

Right
All hand fractures
Left
Ray IV
Ray II
Ray V
Ray III
Ray V
Ray IV
Ray III
Ray II
Ray I
Ray I
n=343
n=312
34 (10%)
136 (40%)
55 (16%)
36 (10%)
64 (19%)
17 (5%)
20 (6%)
5 (1%)
7 (2%)
9 (3%)
6 (2%)
7 (2%)
55 (16%)
36 (10%)
14 (4%)
7 (2%)
15 (4%)
12 (3%)
138 (40%)
61 (18%)
6 (2%)
96 (28%)
5 (1%)
43 (13%)
11 (3%)
11 (3%)
54 (16%)
15 (4%)
2 (1%)
58 (19%)
56 (18%)
42 (13%)
32 (10%)
106 (34%)
16 (5%)
8 (3%)
3 (1%)
10 (3%)
7 (2%)
7 (2%)
45 (14%)
2 (1%)
42 (13%)
18 (6%)
21 (7%)
12 (4%)
19 (6%)
69 (22%)
16 (5%)
26 (8%)
11 (4%)
8 (3%)
10 (3%)
17 (5%)
14 (4%)
16 (5%)
Phalangeal
Phalangeal
Distal
Distal
Middle
Middle
234 (75%)
229 (67%)
147 (47%)
Proximal
Proximal
60 (19%)
Metacarpal
Metacarpal
Carpal
Carpal
1 (0%
18 (6%)
18 (5%)
1 (0%)
3 (1%)
3 (1%)
1 (0%)
9 (3%)
3(1%)
1 (0%)
