# Supplementary material for: Time trends in pediatric hand fracture incidence in Malmö, Sweden, 1950–2016
Source: J Orthop Surg Res. 2021 Apr 9;16:245. doi: 10.1186/s13018-021-02380-y (PMC8034127; doi:10.1186/s13018-021-02380-y)
Supplement: Supplementary file 4 — Additional file 4: Supplement Table 1. Right to left distribution in all hand fractures, phalangeal fractures, and metacarpal/carpal fractures (excluding the scaphoid bone) in all children, and in boys and girls < 16 years in Malmö, Sweden, during 2014–2016. Data are presented as incident rate ratio (IRR) with 95% confidence interval (95% CI). The scaphoid fractures were not examined due to the low number of fractures. [file 13018_2021_2380_MOESM4_ESM.docx]

**Supplement Table 1**

Right to left distribution in all hand fractures, phalangeal fractures, and metacarpal/carpal fractures (excluding the scaphoid bone) in all children, and in boys and girls <16 years in Malmö, Sweden, during 2014–2016. Data are presented as incident rate ratio (IRR) with 95% confidence interval (95% CI). The scaphoid fractures were not examined due to the low number of fractures.

|  | All children | Boys | Girls |
| --- | --- | --- | --- |
| All hand fractures | 1.1 (0.96 to 1.3) | 1.3 (1.05 to 1.6) | 0.9 (0.6 to 1.2) |
| Phalangeal fractures | 1.0 (0.8 to 1.2) | 1.0 (0.8 to 1.3) | 0.9 (0.7 to 1.3) |
| Metacarpal/carpal fractures | 1.8 (1.3 to 2.6) | 2.3 (1.5 to 3.3) | 0.6 (0.2 to 1.7) |
