# Supplementary material for: Time trends in pediatric hand fracture incidence in Malmö, Sweden, 1950–2016
Source: J Orthop Surg Res. 2021 Apr 9;16:245. doi: 10.1186/s13018-021-02380-y (PMC8034127; doi:10.1186/s13018-021-02380-y)
Supplement: Supplementary file 6 — Additional file 6: Supplement Table 3. Unadjusted and age- and sex-adjusted metacarpal/carpal fracture (excluding the scaphoid bone) incidence differences in all children and unadjusted and age-adjusted fracture incidence in boys and girls separately in children < 16 years in Malmö, Sweden, during the years 2014–2016 compared with 1950/1955, 1960/1965, 1970/1975–1979, 1993–1994, and 2005–2006. Data are presented as incident rate ratio (IRR) with 95% confidence interval (95% CI). [file 13018_2021_2380_MOESM6_ESM.docx]

**Supplement Table 3**

Unadjusted and age- and sex-adjusted metacarpal/carpal fracture (excluding the scaphoid bone) incidence differences in all children and unadjusted and age-adjusted fracture incidence in boys and girls separately in children <16 years in Malmö, Sweden, during the years 2014–2016 compared with 1950/1955, 1960/1965, 1970/1975–1979, 1993–1994, and 2005–2006. Data are presented as incident rate ratio (IRR) with 95% confidence interval (95% CI).

|  | Nominator | 2014–2016 | 2014–2016 | 2014–2016 | 2014–2016 | 2014–2016 |
| --- | --- | --- | --- | --- | --- | --- |
|  | Denominator | 1950/1955 | 1960/1965 | 1970/1975–1979 | 1993–1994 | 2005–2006 |
| All children | Unadjusted | 1.2 (0.9 to 1.6) | 0.9 (0.7 to 1.2) | 0.6 (0.5 to 0.7) | 0.6 (0.5 to 0.8) | 0.5 (0.4 to 0.7) |
|  | Age- and sex-adjusted | 1.4 (1.02 to 1.8) | 1.2 (0.9 to 1.6) | 0.7 (0.6 to 0.9) | 0.7 (0.6 to 0.9) | 0.7 (0.6 to 0.9) |
| Boys | Unadjusted | 1.2 (0.9 to 1.7) | 1.1 (0.8 to 1.4) | 0.5 (0.4 to 0.7) | 0.7 (0.5 to 0.9) | 0.5 (0.4 to 0.7) |
|  | Age-adjusted | 1.4 (1.02 to 1.9) | 1.4 (1.02 to 1.8) | 0.7 (0.6 to 0.9) | 0.7 (0.6 to 0.97) | 0.7 (0.5 to 0.9) |
| Girls | Unadjusted | 1.1 (0.5 to 2.2) | 0.6 (0.3 to 1.1) | 0.7 (0.4 to 1.2) | 0.5 (0.3 to 0.97) | 0.7 (0.4 to 1.4) |
|  | Age-adjusted | 1.2 (0.6 to 2.3) | 0.7 (0.4 to 1.3) | 0.8 (0.5 to 1.4) | 0.5 (0.3 to 0.98) | 0.8 (0.4 to 1.6) |
