# Supplementary material for: Time trends in pediatric hand fracture incidence in Malmö, Sweden, 1950–2016
Source: J Orthop Surg Res. 2021 Apr 9;16:245. doi: 10.1186/s13018-021-02380-y (PMC8034127; doi:10.1186/s13018-021-02380-y)
Supplement: Supplementary file 7 — Additional file 7: Supplement Table 4. Phalangeal fracture etiology in Malmö children 0–15 years during six periods; 1950/1955, 1960/1965, 1970/1975–1979, 1993–1994, 2005–2006, and 2014–2016. Etiology is described as trauma activity, trauma mechanism, and trauma severity. Data are presented as proportions (%) of known trauma etiology. [file 13018_2021_2380_MOESM7_ESM.docx]

**Supplement Table 4**

Phalangeal fracture etiology in Malmö children 0–15 years during six periods; 1950/1955, 1960/1965, 1970/1975–1979, 1993–1994, 2005–2006, and 2014–2016. Etiology is described as trauma activity, trauma mechanism, and trauma severity. Data are presented as proportions (%) of known trauma etiology.

|  | **1950/1955** | **1960/1965** | **1970/1975–1979** | **1993–1994** | **2005–2006** | **2014–2016** |
| --- | --- | --- | --- | --- | --- | --- |
| **TRAUMA ACTIVITY** |  |  |  |  |  |  |
| **Known** | **40** | **45** | **56** | **69** | **69** | **75** |
| **Unknown** | **60** | **55** | **44** | **31** | **31** | **25** |
| **Home** | **12** | **9** | **6** | **7** | **2** | **5** |
| **Day nursery** | **0** | **0** | **0** | **2** | **2** | **6** |
| **School** | **6** | **10** | **7** | **4** | **9** | **21** |
| **Work** | **6** | **0** | **0** | **2** | **0** | **0** |
| **Traffic injuries** | **22** | **13** | **14** | **14** | **11** | **6** |
| Bicycle | 18 | 8 | 8 | 9 | 11 | 4 |
| Pedestrian hit by vehicle | 2 | 1 | 1 | 0 | 0 | 0 |
| Moped, motorcycle | 0 | 1 | 1 | 1 | 0 | 0 |
| Car passenger | 2 | 1 | 4 | 1 | 0 | 2 |
| Other | 0 | 2 | 0 | 3 | 0 | 0 |
| **Playing injuries** | **14** | **20** | **13** | **14** | **13** | **14** |
| Playground | 2 | 2 | 1 | 3 | 3 | 1 |
| In-lines, skateboard | 0 | 0 | 1 | 2 | 1 | 2 |
| Sledge, other “snow” | 0 | 0 | 1 | 1 | 1 | 0 |
| Other | 12 | 18 | 10 | 8 | 8 | 11 |
| **Sport injuries** | **33** | **45** | **54** | **49** | **52** | **45** |
| Ball-game | 27 | 38 | 44 | 36 | 42 | 38 |
| Ice-hockey, skating | 2 | 3 | 3 | 1 | 2 | 1 |
| Gymnastics and athletics | 0 | 0 | 1 | 4 | 0 | 2 |
| Horse accidents | 4 | 1 | 3 | 3 | 2 | 0 |
| Wrestling, boxing, etc. "Contact sport" | 0 | 0 | 2 | 4 | 3 | 2 |
| Skiing | 0 | 0 | 3 | 1 | 1 | 0 |
| Other | 0 | 2 | 0 | 1 | 2 | 2 |
| **Fights** | **6** | **3** | **2** | **6** | **10** | **2** |
| **Other** | **0** | **0** | **3** | **2** | **1** | **1** |
| **TRAUMA MECHANISM** |  |  |  |  |  |  |
| **Known** | **84** | **86** | **94** | **92** | **100** | **93** |
| **Unknown** | **16** | **14** | **6** | **8** | **0** | **7** |
| **Falls** | **49** | **49** | **61** | **30** | **37** | **30** |
| On the same plane | 44 | 41 | 53 | 20 | 27 | 22 |
| Between planes | 5 | 8 | 8 | 11 | 10 | 8 |
| **Mechanical force** | **50** | **51** | **39** | **64** | **56** | **68** |
| **Non-classifiable** | **1** | **0** | **0** | **6** | **6** | **3** |
| **TRAUMA SEVERITY** |  |  |  |  |  |  |
| **Known** | **93** | **88** | **96** | **98** | **99** | **95** |
| **Unknown** | **7** | **12** | **4** | **2** | **1** | **5** |
| **Slight** | **66** | **62** | **74** | **78** | **75** | **70** |
| **Moderate** | **9** | **8** | **7** | **18** | **10** | **6** |
| **Severe** | **5** | **1** | **2** | **3** | **0** | **0** |
| **Non-classifiable** | **19** | **29** | **16** | **1** | **15** | **23** |
