# Supplementary material for: Time trends in pediatric hand fracture incidence in Malmö, Sweden, 1950–2016
Source: J Orthop Surg Res. 2021 Apr 9;16:245. doi: 10.1186/s13018-021-02380-y (PMC8034127; doi:10.1186/s13018-021-02380-y)
Supplement: Supplementary file 8 — Additional file 8: Supplement Table 5. Metacarpal/carpal fracture etiology (excluding the scaphoid bone) in Malmö children < 16 years during six periods; 1950/1955, 1960/1965, 1970/1975–1979, 1993–1994, 2005–2006, and 2014–2016. Etiology is described as trauma activity, trauma mechanism, and trauma severity. Data are presented as proportions (%) of known trauma etiology. [file 13018_2021_2380_MOESM8_ESM.docx]

**Supplement Table 5**

Metacarpal/carpal fracture etiology (excluding the scaphoid bone) in Malmö children <16 years during six periods; 1950/1955, 1960/1965, 1970/1975–1979, 1993–1994, 2005–2006, and 2014–2016. Etiology is described as trauma activity, trauma mechanism, and trauma severity. Data are presented as proportions (%) of known trauma etiology.

|  | **1950/1955** | **1960/1965** | **1970/1975–1979** | **1993–1994** | **2005–2006** | **2014–2016** |
| --- | --- | --- | --- | --- | --- | --- |
| **TRAUMA ACTIVITY** |  |  |  |  |  |  |
| **Known** | **44** | **65** | **65** | **79** | **67** | **75** |
| **Unknown** | **56** | **35** | **35** | **21** | **33** | **25** |
| **Home** | **7** | **2** | **2** | **7** | **0** | **2** |
| **Day nursery** | **0** | **0** | **0** | **0** | **1** | **2** |
| **School** | **25** | **10** | **5** | **6** | **13** | **17** |
| **Work** | **0** | **0** | **0** | **0** | **0** | **0** |
| **Traffic injuries** | **18** | **20** | **14** | **20** | **9** | **11** |
| Bicycle | 18 | 7 | 10 | 15 | 8 | 10 |
| Pedestrian hit by vehicle | 0 | 10 | 0 | 0 | 0 | 0 |
| Moped, motorcycle | 0 | 3 | 3 | 2 | 1 | 1 |
| Car passenger | 0 | 0 | 0 | 1 | 0 | 0 |
| Other | 0 | 0 | 0 | 1 | 0 | 0 |
| **Playing injuries** | **11** | **25** | **15** | **18** | **5** | **18** |
| Playground | 0 | 2 | 2 | 1 | 1 | 3 |
| In-lines, skateboard | 0 | 0 | 1 | 2 | 3 | 4 |
| Sledge, other “snow” | 0 | 0 | 2 | 1 | 0 | 3 |
| Other | 11 | 24 | 10 | 13 | 1 | 9 |
| **Sport injuries** | **18** | **15** | **24** | **25** | **29** | **35** |
| Ball-game | 11 | 10 | 9 | 8 | 13 | 21 |
| Ice-hockey, skating | 4 | 2 | 7 | 7 | 3 | 1 |
| Gymnastics and athletics | 0 | 0 | 0 | 1 | 0 | 1 |
| Horse accidents | 4 | 0 | 3 | 2 | 2 | 2 |
| Wrestling, boxing, etc. "Contact sport" | 0 | 2 | 1 | 2 | 5 | 8 |
| Skiing | 0 | 2 | 4 | 4 | 3 | 3 |
| Other | 0 | 0 | 0 | 0 | 3 | 0 |
| **Fights** | **18** | **27** | **38** | **24** | **42** | **12** |
| **Other** | **4** | **0** | **2** | **0** | **0** | **4** |
| **TRAUMA MECHANISM** |  |  |  |  |  |  |
| **Known** | **84** | **98** | **96** | **96** | **100** | **96** |
| **Unknown** | **16** | **2** | **4** | **4** | **0** | **4** |
| **Falls** | **63** | **76** | **82** | **45** | **40** | **44** |
| On the same plane | 48 | 63 | 70 | 26 | 30 | 31 |
| Between planes | 15 | 13 | 12 | 18 | 10 | 13 |
| **Mechanical force** | **37** | **22** | **18** | **48** | **56** | **52** |
| **Non-classifiable** | **0** | **1** | **0** | **8** | **4** | **4** |
| **TRAUMA SEVERITY** |  |  |  |  |  |  |
| **Known** | **84** | **98** | **97** | **99** | **99** | **100** |
| **Unknown** | **16** | **2** | **3** | **1** | **1** | **0** |
| **Slight** | **70** | **75** | **83** | **78** | **86** | **63** |
| **Moderate** | **17** | **9** | **11** | **17** | **11** | **11** |
| **Severe** | **2** | **11** | **3** | **5** | **0** | **1** |
| **Non-classifiable** | **11** | **4** | **3** | **0** | **2** | **25** |
