# Supplementary material for: Time trends in pediatric hand fracture incidence in Malmö, Sweden, 1950–2016
Source: J Orthop Surg Res. 2021 Apr 9;16:245. doi: 10.1186/s13018-021-02380-y (PMC8034127; doi:10.1186/s13018-021-02380-y)
Supplement: Supplementary file 8 [file 13018_2021_2380_MOESM3_ESM.docx]

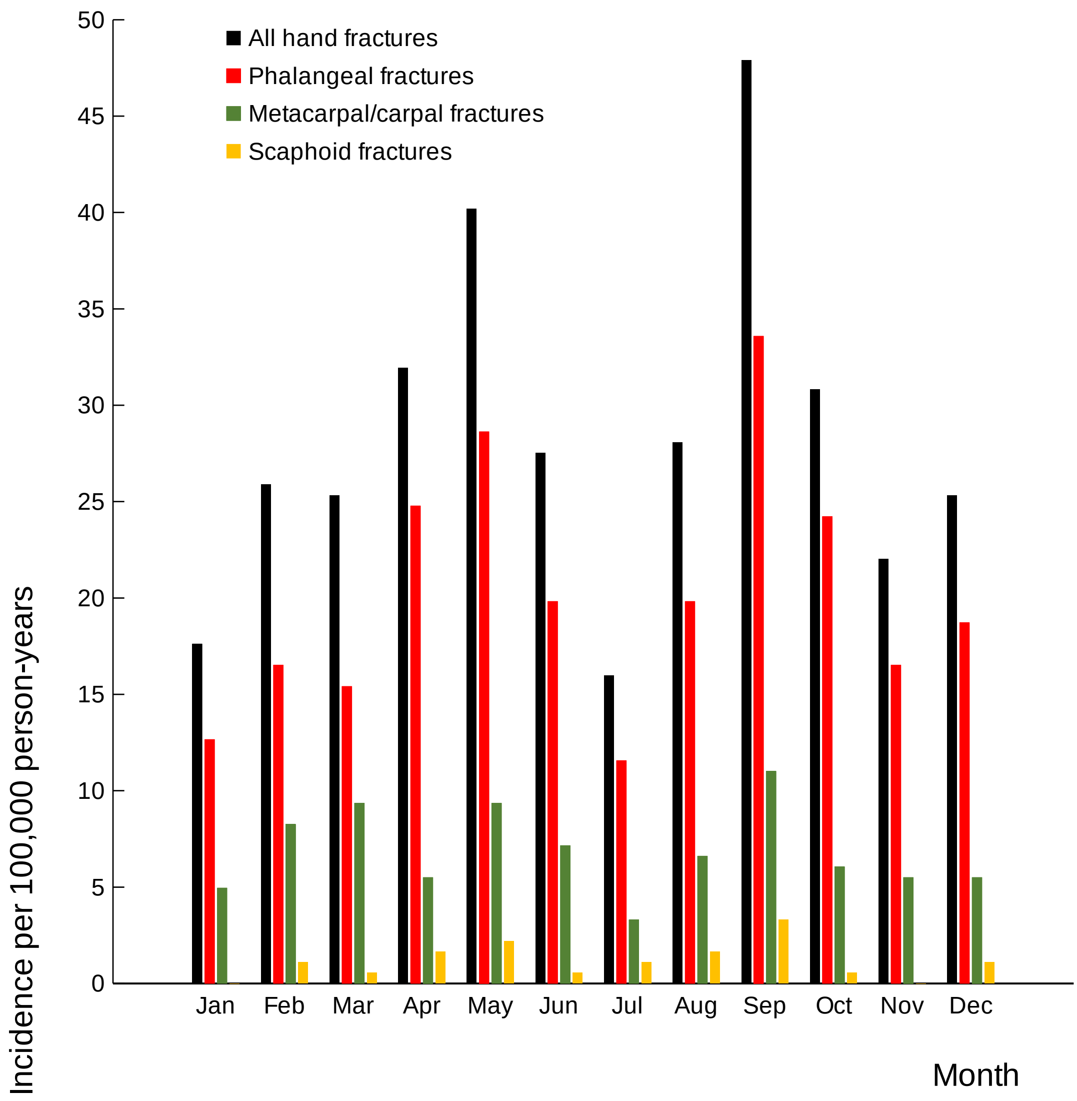

All hand fractures
Phalangeal fractures
Metacarpal/carpal fractures
Scaphoid fractures
Incidence per 100,000 person-years
0
5
10
15
20
25
30
35
40
45
50
Jan
Feb
Mar
Apr
May
Jun
Jul
Aug
Sep
Oct
Nov
Dec
Month
